# Supplementary material for: Cardiac and Locomotor Responses to Acute Stress in Signal Crayfish Pacifastacus leniusculus Exposed to Methamphetamine at an Environmentally Relevant Concentration
Source: Int J Environ Res Public Health. 2020 Mar 21;17(6):2084. doi: 10.3390/ijerph17062084 (PMC7143509; doi:10.3390/ijerph17062084)
Supplement: Supplementary file 1 [file ijerph-17-02084-s001.pdf]

Supplementary materials

Table S1. Mean heart rate (beats min^{-1}) of individual METH-exposed signal crayfish over 30 min pre- and post-exposure to stress stimulus. Dark grey indicates significant increase, light grey indicates significant decrease, and white indicates no significant difference in cardiac activity ($\alpha = 0.05$).

Crayfish	Stress	Day 0	Exposure days				Depuration days			
			1	7	14	21	1	2	7	14
1	Pre	56.7	67.7	70.3	61.5	51.7	52.6	58.9	52.2	51.6
	Post	67.6	86.7	58.5	55.4	60.6	50.7	55.7	50.9	57.1
2	Pre	97.4	81.2	68.5	88.0	73.7	69.5	63.9	61.4	70.9
	Post	101.6	87.6	82.0	87.0	73.6	68.1	65.6	73.0	71.7
3	Pre	96.1	72.0	48.0	61.9	93.5	58.4	67.2	66.0	54.2
	Post	101.7	79.7	68.4	54.4	84.0	58.7	60.8	69.8	53.8
4	Pre	55.7	43.0	50.5	47.4	45.4	43.6	50.2	32.0	36.6
	Post	53.4	41.5	59.4	49.4	52.2	44.4	45.6	33.8	36.7
5	Pre	53.2	55.3	72.2	53.8	93.1	53.7	57.2	52.6	49.3
	Post	65.4	66.6	56.6	54.4	67.4	58.8	67.1	53.9	54.1
6	Pre	76.2	68.9	59.2	54.7	61.4	57.1	61.2	61.3	56.3
	Post	80.5	64.4	59.2	63.3	64.3	65.8	61.2	58.8	65.2
7	Pre	71.3	66.3	46.6	48.3	44.0	42.5	40.2	65.0	48.3
	Post	67.7	52.6	58.6	63.0	47.4	43.8	44.1	61.2	66.0
8	Pre	58.6	58.0	55.8	48.6	45.3	43.6	41.0	48.3	75.6
	Post	62.1	58.6	65.8	54.6	52.2	40.9	45.0	48.6	77.6
9	Pre	93.5	57.6	70.6	63.1	68.6	87.7	39.6	47.3	42.6

	Post	67.5	57.7	59.7	64.9	67.7	84.9	38.7	47.5	43.2
10	Pre	64.3	57.9	57.5	65.4	82.6	83.1	77.7	79.8	77.9
	Post	82.2	74.6	77.1	67.8	79.7	80.9	79.3	80.7	80.0
11	Pre	58.2	88.1	66.6	58.5	59.5	61.3	56.1	64.8	56.0
	Post	60.3	76.5	64.8	61.8	60.7	61.5	53.6	66.3	53.6
12	Pre	83.9	47.7	45.0	50.3	54.6	47.9	58.9	76.0	59.0
	Post	59.3	66.1	41.1	60.8	60.6	61.4	55.7	76.7	68.8

Table S2. Mean heart rate (beats min^{-1}) of individual unexposed control signal crayfish 30 min pre- and post-exposure to the stress stimulus. Dark grey indicates significant increase, light grey indicates significant decrease (α = 0.05), and white indicates no significant difference of cardiac activity. M, moulted.

Crayfish	Stress	Day 0	Exposure days				Depuration days			
			1	7	14	21	1	2	7	14
1	Pre	57.3	52.8	55.9	69.1	67.0	59.0	52.2	60.4	62.2
	Post	71.8	61.2	70.7	74.7	72.5	63.0	58.5	79.2	64.6
2	Pre	67.9	56.4	70.5	58.1	70.3	64.1	59.6	59.5	60.9
	Post	72.7	71.1	82.5	69.3	75.9	81.7	62.8	82.5	62.7
3	Pre	96.9	55.2	80.9	84.2	111.0	72.5	101.8	75.2	70.6
	Post	93.6	46.6	94.4	85.5	105.5	95.6	88.7	76.1	70.9
4	Pre	71.7	70.8	64.8	54.2	59.0	54.0	67.4	68.6	69.6
	Post	72.1	74.1	65.3	54.6	73.2	58.5	73.2	69.8	70.9
5	Pre	53.2	53.0	60.6	62.9	64.1	52.8	51.7	49.4	49.2
	Post	54.4	49.5	89.0	79.8	61.8	61.5	52.0	64.8	53.7
6	Pre	84.9	76.6	68.7	83.1	73.1	78.7	76.0	46.2	49.5
	Post	80.5	96.2	96.4	93.5	75.8	91.0	76.0	61.6	60.5
7	Pre	49.2	49.9	52.2	56.0	79.2	47.5	44.1	52.5	44.2
	Post	49.0	72.9	70.4	63.2	60.7	55.9	46.9	63.9	50.4
8	Pre	54.7	47.7	60.3	63.8	82.8	54.8	64.2	63.7	62.6
	Post	55.9	49.8	89.0	85.4	72.1	61.7	48.5	81.7	61.9
9	Pre	48.1	56.9	50.2	58.5	69.7	55.2	49.9	61.8	58.2
	Post	54.3	59.2	62.4	57.0	66.4	60.7	52.5	57.7	71.6
10	Pre	45.4	54.0	67.1	52.0	62.6	51.3	49.5	50.8	46.3

	Post	44.1	67.3	87.5	62.7	57.0	63.3	48.2	54.0	60.0
11	Pre	75.0	57.1	65.4	71.2	78.7	56.7	70.7	60.7	59.6
	Post	68.6	74.1	101.5	68.0	72.8	66.5	58.6	68.8	78.8
12	Pre	49.0	43.0	49.9	49.2	58.0	54.1	46.9	47.1	52.6
	Post	47.9	46.0	76.4	51.4	56.2	63.5	49.2	56.6	62.4
